# Ischemic Heart Disease and Chronic Obstructive Pulmonary Disease Hospitalizations in Japan Before and After the Introduction of a Heated Tobacco Product

**DOI:** 10.3389/fpubh.2022.909459

**Published:** 2022-06-28

**Authors:** Angela van der Plas, Meagan Antunes, Alba Romero-Kauss, Matthew Hankins, Annie Heremans

**Affiliations:** PMI R&D, Philip Morris Products S.A., Neuchâtel, Switzerland

**Keywords:** real-world data, heated tobacco, hospitalizations, time-trend analysis, COPD, IHD

## Abstract

To substantiate the beneficial effects of switching from cigarette smoking to heated tobacco products (HTP), this study conducted a time-trend analysis using data from the Japanese Medical Data Center (JMDC) database. Specifically, we assessed hospitalization numbers for chronic obstructive pulmonary disease (COPD) exacerbations and acute ischemic heart disease (IHD) before and after the introduction of HTPs in the Japanese market. This study replicated a previous study using a different Japanese real-world data source (Medical Data Vision). We retrieved the number of hospitalizations associated with the International Classification of Diseases-10 codes for COPD and IHD from 2010 to 2019−5 years before to 4 years after introducing HTPs in the Japanese market—from the JMDC database. Then, we used interrupted time-series analyses to test the hypothesis that the introduction of HTPs is associated with a reduction in hospitalizations for COPD (all codes), COPD exacerbation, COPD exacerbation plus lower respiratory tract infections (LRTI), and IHD, adjusting for age, sex, seasonality, and flu vaccination rates. Analysis of all available data from the JMDC database revealed a significant reduction in the number of hospitalizations for COPD (all codes; *P* = 0.0001) and IHD using Diagnosis Procedure Combination data on causative disease flags (*P* < 0.00001). We also observed a non-significant reduction in hospitalizations for COPD plus LRTI as well as IHD after HTP introduction in Japan. This study confirmed the findings of our previous study where a decrease in hospitalizations due to COPD exacerbation after the introduction of HTPs in Japan was also shown. Nevertheless, these findings warrant further research to evaluate the impact of HTPs on the health of populations in other countries where these products have been introduced.

## Introduction

To contrast the harmful effects of cigarette smoking, new, smoke-free, products have been developed to replace regular cigarettes with less harmful alternative products for smokers who would otherwise continue smoking. One such product is the Tobacco Heating System, marketed as *IQOS*^*TM*^. It was first launched in a city pilot in Japan in November 2014 and has been the most prevalent heated tobacco product (HTP) in Japan. It is currently marketed in over 50 countries. In July 2020, the US Food and Drug Administration authorized the commercialization of *IQOS* as a modified risk tobacco product under a reduced exposure order (1).

The use of real-world data (RWD) is a viable option to gather evidence related to tobacco products, and time-trend analyses have been used to assess the impact of population-level interventions such as smoking bans and their effects on smoking-related diseases and their endpoints (2–4). RWD are available in Japan through multiple sources. As the first real-world evidence (RWE) study, Philip Morris International (PMI) conducted (manuscript in press) a time-trend analysis on hospitalizations for chronic obstructive pulmonary disease (COPD) and ischemic heart disease (IHD) among the Japanese population before and after the introduction of HTPs; this study used RWD retrieved from the Medical Data Vision (MDV) database. Results from this first RWE study showed a statistically significant decrease in the number of hospitalizations due to COPD exacerbation (*P* < 0.01) after the introduction of HTPs. To confirm the findings of the MDV study, we replicated the same analysis using data from another large Japanese database, the Japanese Medical Data Center (JMDC) database. The aim was to assess the number of hospitalizations due to COPD, COPD exacerbation, COPD exacerbations plus lower respiratory tract infection (LRTI), and IHD before and after introduction of HTPs in the Japanese market. This study in conjunction with the previous Japanese data analysis shed light on the possible impact of population-based interventions and the health of the population in the absence of long-term subject specific data.

## Materials and Methods

### Data Source

This study was conducted in accordance with the Guidelines for Good Epidemiological Practice. The data were retrieved from the JMDC database. JMDC contains accumulated receipts (inpatient, outpatient, and dispensing) and Diagnosis Procedure Combination (DPC) data of ~7.3 million patients (as of April 2020) (5). The database encrypts all personal information to a high level with irreversible anonymization technology.

The study population consisted of adults (aged ≥20–74 years) who were hospitalized between January 2010 and December 2019. JMDC does not include data on patients aged 75 years and older because those patients are automatically enrolled in a state-run insurance system. Among all hospitalizations, the selected endpoints were monthly hospitalizations due to COPD (for all related International Classification of Diseases-10 codes), COPD exacerbation, COPD exacerbation plus LRTI, and IHD. Analyses were conducted using both all claims data (broad definition) as well as only DPC data (strict definition).

Because claims data were anonymized before they were received, we waived individual participants' consent according to the Ethical Guidelines for Medical and Health Research Involving Human Subjects (6).

### Statistical Analyses

This study assessed the intervention of HTPs' introduction in the Japanese market, which was informed by the level of market penetration of HTP sales. In Japan, HTPs were marketed in stages from 2014 (single city pilot) to 2016 (national launch of *IQOS*). Recent independent analyses have shown that the introduction of *IQOS* has accelerated the decline in cigarette sales in Japan since 2016 (1, 7). Further, a data source provided by PMI indicated that the market share of HTPs in Japan increased from 0.01% in January 2015 to about 7% in January 2017. From this point, the market share started increasing more rapidly, reaching 25% by the end of 2019. Thus, January 2017 was chosen as the starting point of the intervention, which was measured using a binary variable with a value of 0 or 1 for the pre- or post-HTP period, respectively.

We conducted an interrupted time-series analysis (ITS) to assess the association between the endpoints and the intervention. ITS is an appropriate quasi-experimental approach when a randomized trial is not feasible; recently, studies have used it to evaluate public health interventions. To implement this statistical design, we applied quasi-Poisson regression using the logarithm link function. Contrary to the Poisson distribution, the quasi-Poisson model allows the variance to be proportional rather than equal to the mean, which is better suited to this analysis. The model included at least the time, intervention, and their interaction as covariates, whose regression coefficients estimate the pre-intervention slope, the change in level at the intervention point (step change), and the change in slope from pre-intervention to post-intervention (trend change), respectively. Other confounders considered were average age, sex distribution (percentage of women), seasonality, and annual rate of influenza vaccination. To test for autocorrelation, the plot of the residuals and autocorrelation and partial autocorrelation function were visually examined. Since the JMDC database includes relatively few patient records for the years before 2013, we conducted the main analyses using data for 2013–2019. To test the robustness of the analyses, we performed sensitivity analyses using different breakpoints (January 2016 and January 2018) using the full data for 2010–2019 and using only DPC data for 2013–2019 (strict definition).

Table 1 provides an overview of the specifications for the regression models considered for this analysis.

**Table 1:** Overview of interrupted time-series model specifications.

**Explaining variables**	**1**	**2**	**3**	**4**
	**Lean model**	**Age + sex**	**Age + sex + season**	**Age + sex + season + flu vaccination**
Intercept	X	X	X	X
Time	X	X	X	X
Women%		X	X	X
Mean age		X	X	X
HTP = intervention dummy	X	X	X	X
HTP ^*^ Time (interaction)	X	X	X	X
Flu vaccination				X
Seasonal dummy (summer = reference)				
Spring			X	X
Autumn			X	X
Winter			X	X

*HTP, heated tobacco product*.

* *Indicates interaction term*.

To further examine the effects of seasonality on the hospitalization trends, time series decomposition was performed. Using an additive decomposition model, the original time series was split into seasonal, trend, and random components. The de-seasonalized trend was obtained by subtracting the seasonal component from the original trend and was then compared to the results of the ITS analysis.

## Results

Between 2010 and 2019, the total number of hospitalizations increased 8.42 times (from 53,481 in 2010 to 450,761 in 2019). The yearly rate of increase was the highest from 2012 to 2013 (48.98%), followed by 2011 to 2012 (43.01%) and 2014 to 2015 (39.44%). After 2015, the average rate of increase was 17.05% per year.

**Figure 1:**
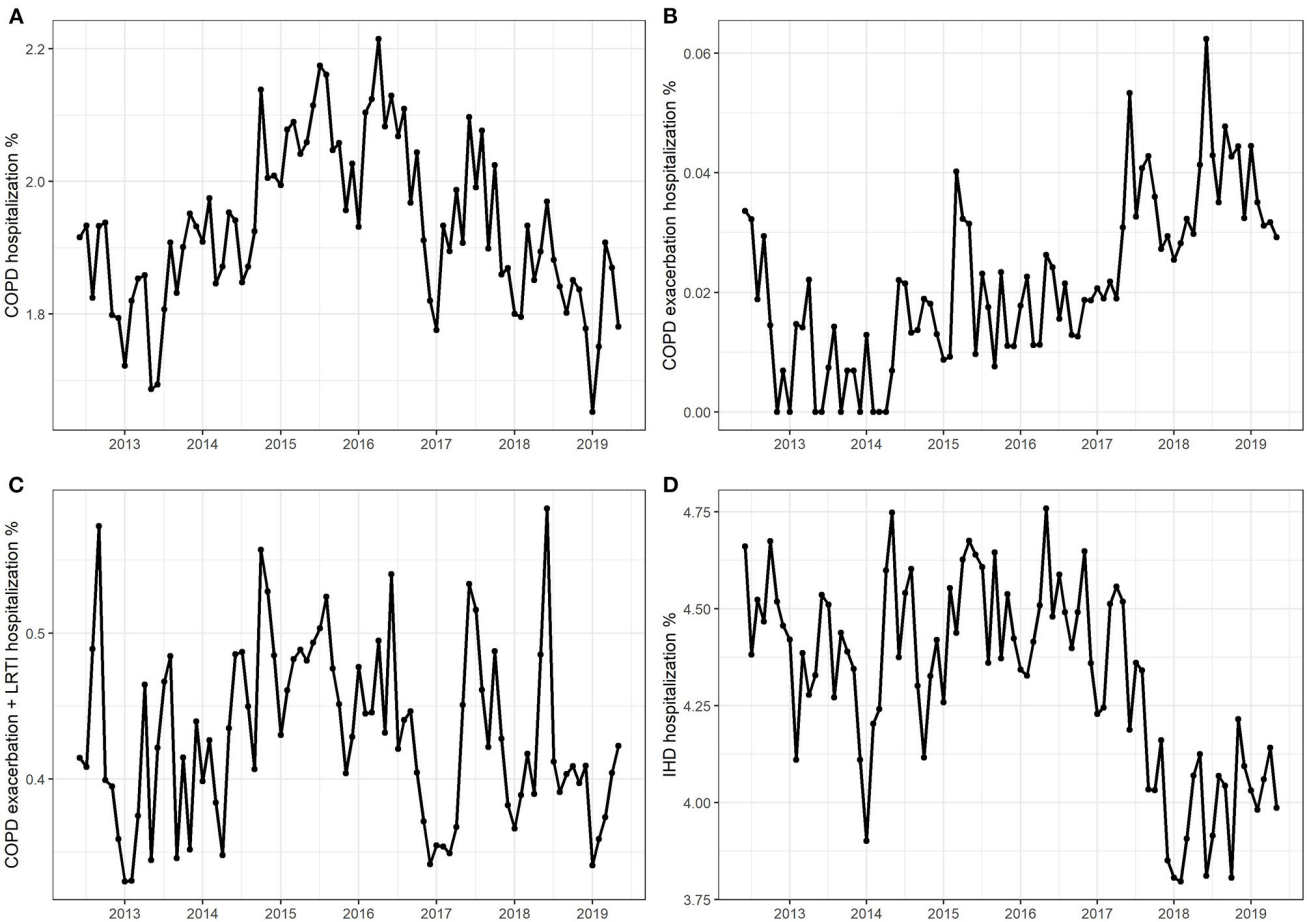
Monthly hospitalization numbers due to **(A)** chronic obstructive pulmonary disease (COPD), **(B)** COPD exacerbations, **(C)** COPD exacerbations + lower respiratory tract infection, and **(D)** ischemic heart disease.

The average number of hospitalizations due to COPD amounted to 1.93% of total hospitalizations, with a fluctuating trend starting from 1.83% in 2013, increasing to 2.08% in 2016, and then decreasing to 1.82% in 2019 (Figure 1A). Hospitalization numbers due to COPD exacerbations plus LRTI showed a similar pattern, increasing from 0.40% in 2013 to 0.48% in 2015, and then decreasing to 0.41% in 2019, with an average of 0.43% (Figure 1C). Hospitalization numbers due to COPD exacerbation presented an increasing trend with an average of 0.02%, reaching a maximum of 0.04% in 2019 (Figure 1B). The average number of hospitalizations due to IHD was 4.32%, decreasing from 4.49% in 2016 to 4.02% in 2019 (Figure 1D).

The number of hospitalizations for all endpoints were lower in women than men. Men had double the number of hospitalizations due to COPD than women (men, 2.59%; women, 1.34%). For COPD exacerbation, the number of hospitalizations for men were five times higher (men, 0.037%; women, 0.007%). For COPD exacerbation plus LRTI, men had 2.5 times more hospitalizations (men, 0.61%; women, 0.21%). For IHD, men had 3.7 times more hospitalizations (men, 7.1%; women, 1.9%). Moreover, in all four patient cohorts, the 45–69-year age group accounted for the most cases (>60%), with the highest proportions of hospitalizations being for COPD exacerbation (63–91%) and IHD (77–81%).

**Figure 2:**
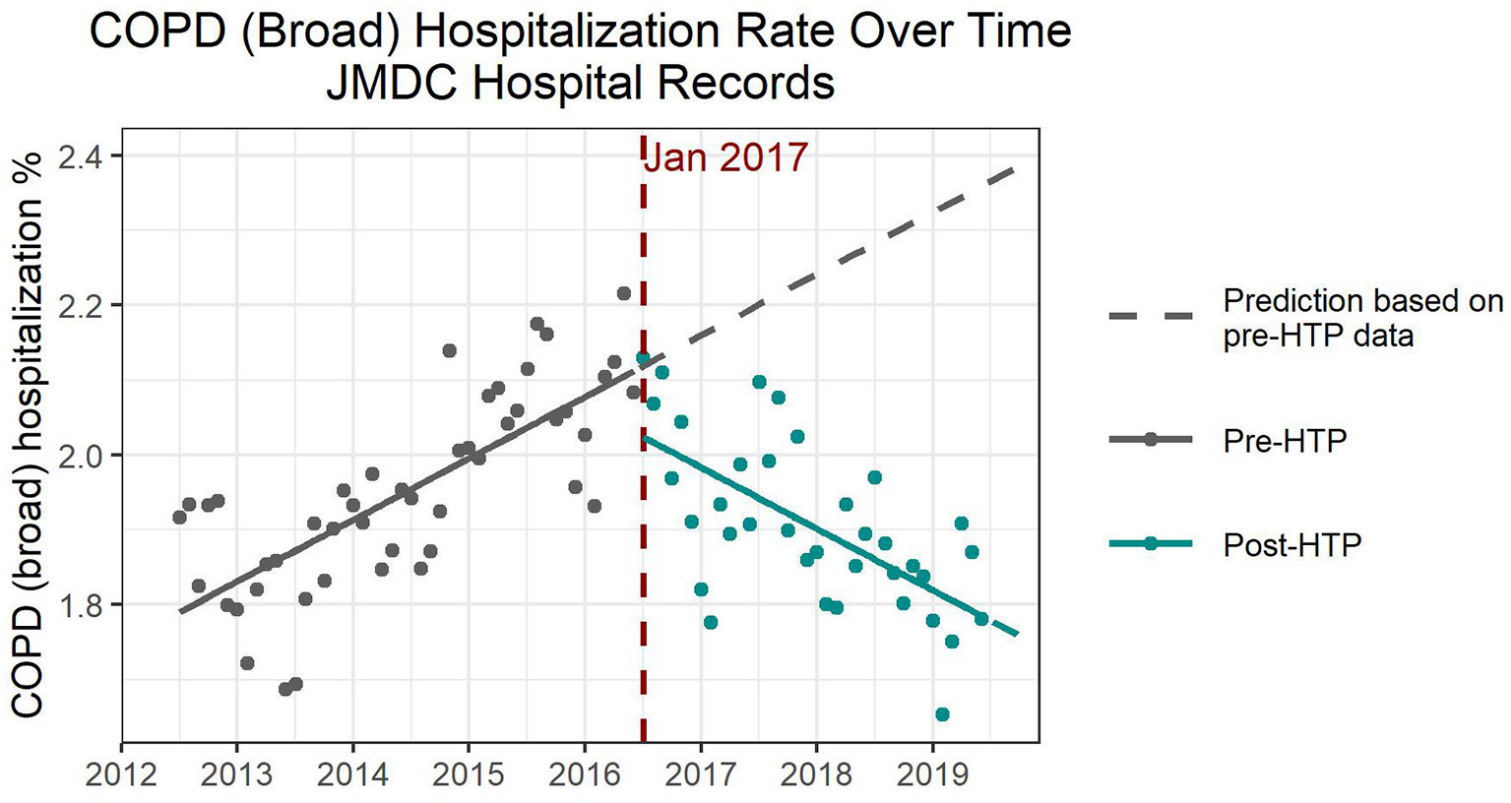
Expected and observed trends in hospitalization numbers due to chronic obstructive pulmonary disease after introduction of heated tobacco products in Japan.

For COPD (all codes), hospitalization numbers dropped remarkably by 0.1–0.2% when comparing the pre- and post-HTP introduction time trends. In all four models, pre-HTP COPD hospitalization numbers showed an increasing trend. ITS models with and without adjustment for confounders consistently showed that the introduction of HTP coincided with a significant decrease in the number of hospitalizations immediately following the intervention, followed by a significant decreasing trend in COPD hospitalizations (Figure 2 and Supplementary Table 1). According to the ITS models adjusted for confounders, increasing the percentage of women and average age of the population would significantly increase the number of COPD hospitalizations. Further, autumn, winter, and spring saw significantly higher hospitalization numbers due to COPD than summer, whereas annual flu vaccination rate did not significantly affect COPD hospitalization numbers.

For COPD exacerbations, there was an increase in hospitalization numbers between the pre- and post-HTP trends. However, the absolute counts were extremely low for this endpoint, with a maximum of only 179 hospitalizations observed in 2019. The ITS models for COPD exacerbation revealed an immediate increase, and an acceleration in the increasing trend, following the introduction of HTPs; however, neither effect was statistically significant (Supplementary Table 2). Sex, age, and annual flu vaccination were all insignificant as confounders. Winter saw significantly higher hospitalization numbers due to COPD exacerbations than summer, but we detected no difference for autumn or spring. However, because of low event counts for COPD exacerbations, the results of this analysis must be interpreted with caution.

Hospitalization numbers for COPD exacerbations plus LRTI showed a decrease of 0.03–0.04% between the pre- and post-HTP introduction time trends. The results revealed an acceleration in the reduction of COPD exacerbations plus LRTI hospitalizations following HTP introduction (Supplementary Table 3). However, this trend change is only significant when cofounders were not taken into account. Although there was an immediate decrease in hospitalizations in all four models, it was not significant. Mean age was significant as a confounder, with hospitalization numbers increasing with average age. Moreover, sex became significant after adjusting for seasonality, with an increasing percentage of women resulting in increased hospitalization numbers. Winter and spring saw significantly higher hospitalization numbers than summer, but there was no significant difference for autumn. Annual influenza vaccination rates did not have a significant effect on hospitalization numbers.

On average, there was a lower proportion of hospitalizations due to IHD in the post-HTP years compared to the pre-HTP years. The decrease amounted to 0.28% when considering the 2017–2019 period and 0.41% if only the 2018–2019 period was compared with the pre-HTP period. The pre-HTP trend was increasing without adjusting for covariates; however, it decreased after adjusting for other covariates, indicating that the model fitting was sensitive to the covariates (Supplementary Table 4). For three of the four models, there was a significant positive effect in the level associated with the intervention, leading to an upward spike at the time of HTP introduction. This was followed by an acceleration in the reduction of hospitalizations; however, the trend change was only significant without adjusting for confounders. Age was a significant confounder, with an increase in average age resulting in higher hospitalization numbers. Sex, seasonality, and flu vaccination were all insignificant as confounders.

### Sensitivity Analyses

Results of the sensitivity analysis using 2016 as the breakpoint were consistent with the main analysis for all endpoints. Results of the sensitivity analysis using 2018 as the breakpoint were similar to the main analysis for COPD, with a significant decreasing trend in hospitalizations following the intervention. This later breakpoint, however, resulted in a decreasing pre-HTP trend, as opposed to the increasing trend observed in the main analysis, causing the negative step change immediately following the intervention to be less pronounced. Further, applying the later breakpoint for COPD exacerbations resulted in a decreasing trend following HTP introduction, contrary to the upward trend observed in the main analysis. However, again, due to small event counts, results for this endpoint must be interpreted with caution. For COPD exacerbations plus LRTI, choosing a later breakpoint changed the relative size of the level and interaction effect. Although the main analysis showed a clear downward shift in the levels, this was not observed when applying the shorter post-HTP time frame. Nevertheless, the effect on the slope tended to be more pronounced in this sensitivity analysis. Moreover, results for IHD were similar to the main analysis, with an acceleration in the decreasing trend post-HTP introduction. However, the positive step change detected in the main analysis was not present when considering the later breakpoint.

Finally, the sensitivity analysis using the full-length data (2010–2019) did not show significant differences compared with the main analysis results.

### Strict Definition

For the sensitivity analyses using the strict definition of the study endpoints, we defined hospitalizations as the number of hospitalizations due to each disease endpoint, with the causative flag divided by the total number of DPC hospitalization claims. There were 50–60% total DPC hospitalization claims among the overall hospitalization claims. Further, the number of hospitalizations due to COPD according to the strict definition was <2% of that by the broad definition, while hospitalizations due to IHD were about 30–40% of those by the broad definition. This sensitivity analysis used the study period of 2013–2019 and the breakpoint of January 2017.

Before the introduction of HTPs, hospitalization numbers due to COPD revealed a slight increasing (decreasing) trend before (after) adjusting for confounders. All four models showed a slight increase in hospitalizations immediately following the intervention, as well as a decreasing trend in the post-HTP period; however, neither effect was statistically significant. According to Model 2 (Supplementary Table 1), increasing the average age would result in an increase in COPD hospitalizations. However, this effect became insignificant after adjusting for seasonality. The percentage of women and the annual influenza vaccination rate were not significant as confounders. Further, none of the seasons showed significant differences in hospitalization numbers compared with the reference season of summer.

The overall trend of COPD exacerbations was increasing, especially from 2018 onward. However, there was an immediate drop after HTP introduction in all four models; this effect was not significant in Model 1 but became significant after adjusting for confounders (Supplementary Table 2). Following HTP introduction, we observed an acceleration in the increasing trend of hospitalizations for COPD exacerbation in all four models, but it was not significant. Sex, age, seasonality, and flu vaccination were all insignificant as confounders. These results must be interpreted with caution because of the low number of hospitalizations due to COPD exacerbation.

Similar to when using the models for COPD exacerbation, the overall trend of hospitalization due to COPD exacerbation plus LRTI was increasing, especially from 2018 onward. There was an immediate drop after HTP introduction, followed by an increasing trend, in all four models, but neither effect was significant (Supplementary Table 3). As with the COPD exacerbation endpoint, sex, age, seasonality, and flu vaccination were all insignificant as confounders.

**Figure 3:**
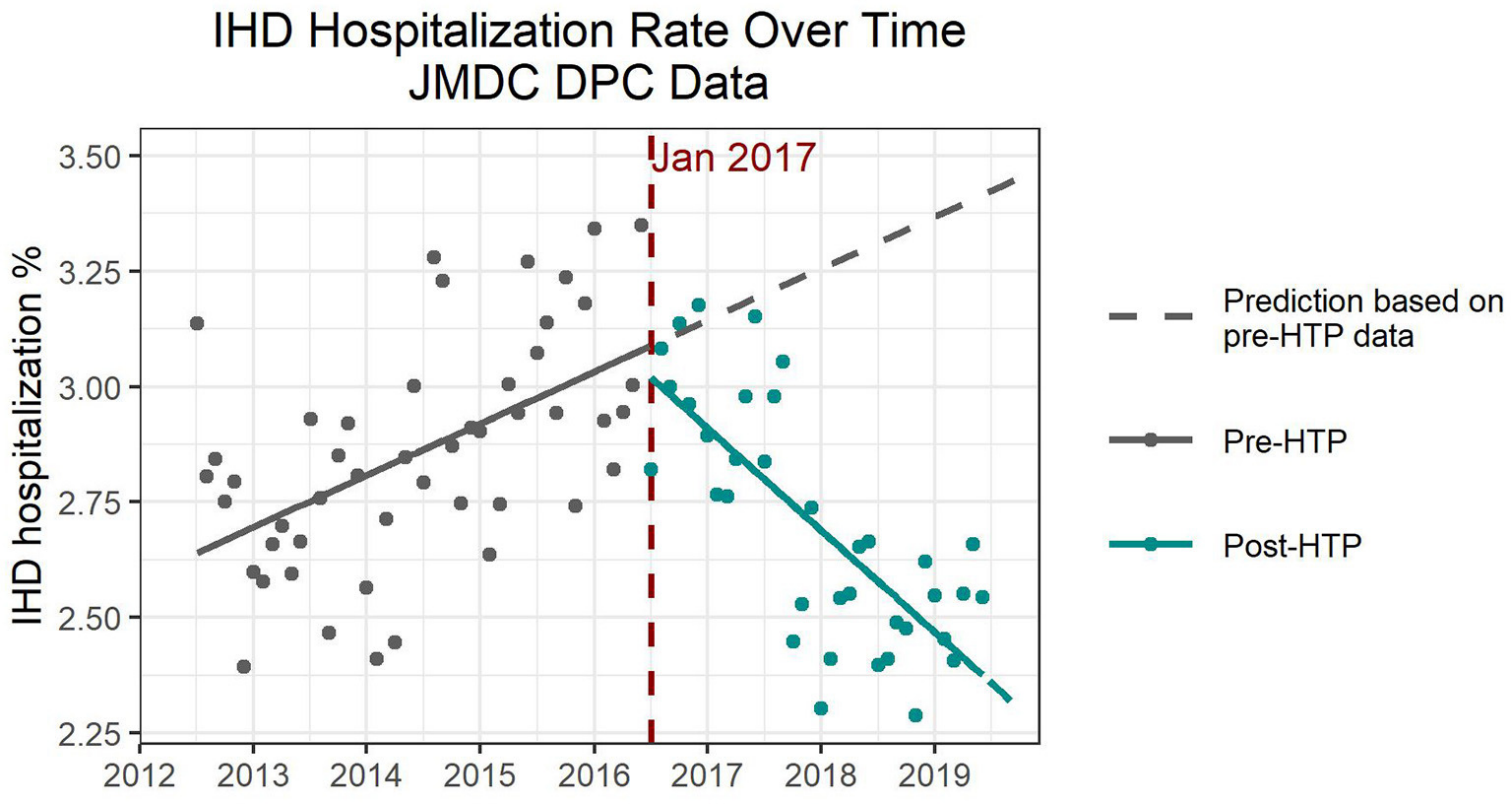
Expected and observed trends in hospitalization numbers due to ischemic heart disease after introduction of heated tobacco products in Japan, using only Diagnosis Procedure Combination data.

Hospitalization numbers due to IHD increased over time prior to the introduction of HTPs, followed by a reversal and decreasing trend after HTP introduction (Figure 3). All four models indicated a slight increase shortly after introducing HTPs, followed by an acceleration of the decreasing trend, both of which were significant (Supplementary Table 4). Based on Model 2, both increasing the women's percentage and average age would increase the number of hospitalizations due to IHD; however, only average age was significant. There were no significant differences in hospitalization numbers due to IHD between the seasons. As with the previous endpoints, flu vaccination rate was also insignificant as a confounder for IHD hospitalization.

### Time Series Decomposition

The results of the time series decomposition were consistent with the results of the ITS analyses for all endpoints. The trend components for COPD (all codes), COPD exacerbation + LRTI and IHD all showed a decrease in hospitalizations following HTP introduction in January 2017. The trend component for COPD exacerbation showed an increasing trend, which was again impacted by the low absolute counts for this endpoint. Further, the seasonal components for all endpoints showed increased hospitalization rates in winter compared to summer, which is also consistent with the results observed from the ITS analysis.

**Figure 4:**
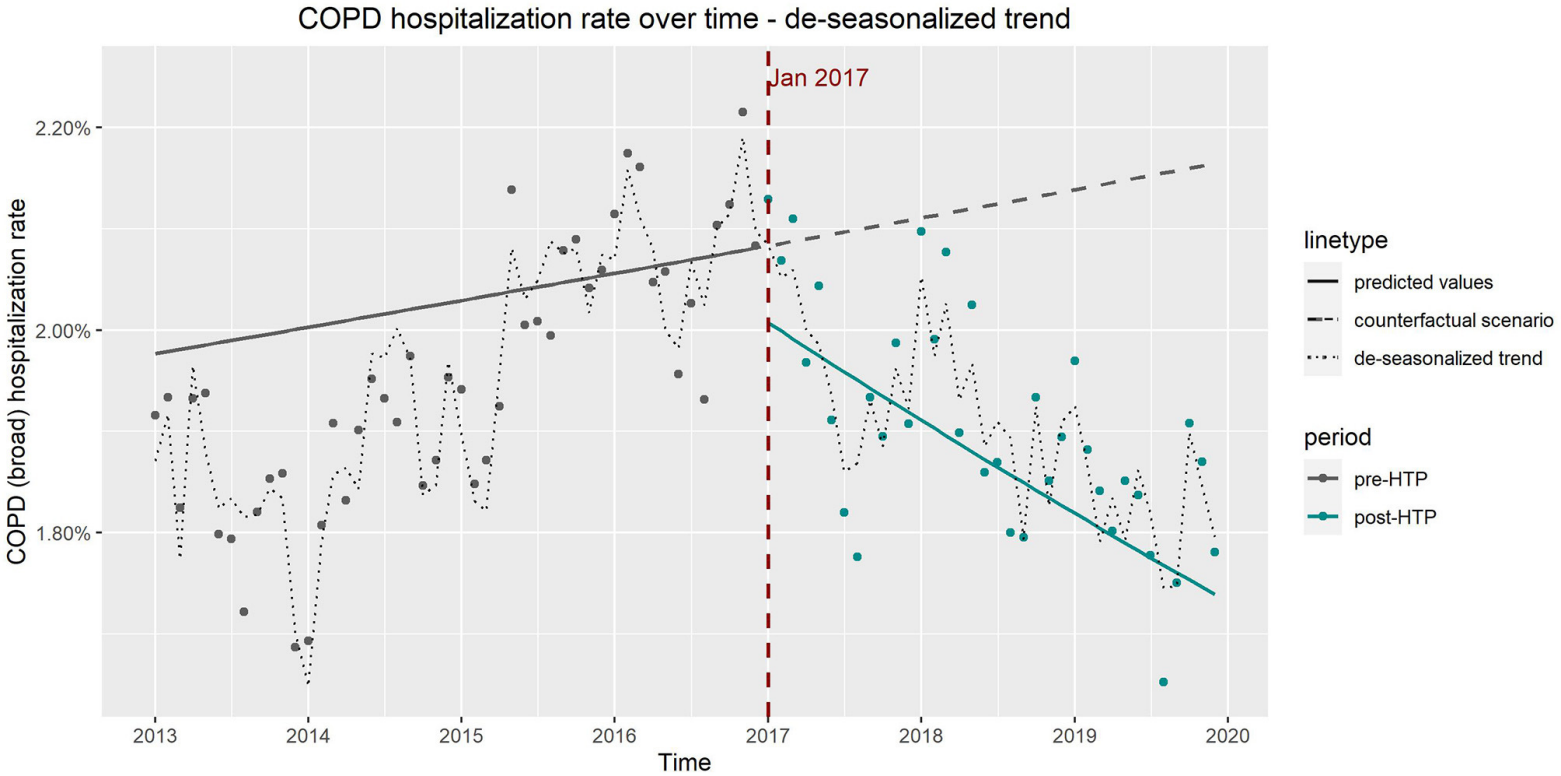
Observed vs. expected trends in hospitalizations due to chronic obstructive pulmonary disease from results of interrupted time series model which includes age, sex, and seasonality as covariates and de-seasonalized time trend.

**Figure 5:**
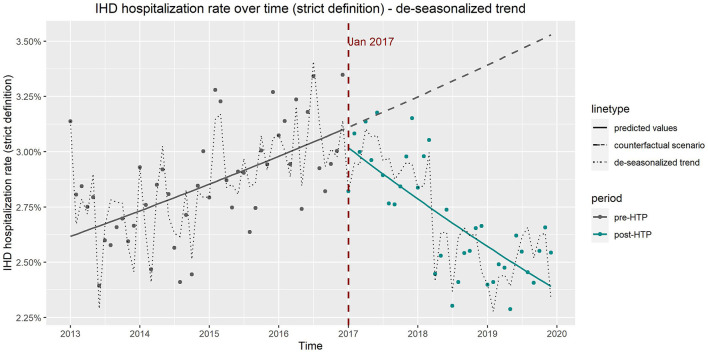
Observed vs. expected trends in hospitalizations due to ischemic heart disease from results of interrupted time series model which includes age, sex, and seasonality as covariates and de-seasonalized time trend, using only Diagnosis Procedure Combination data.

The de-seasonalized trends obtained by subtracting the seasonal component from the original trend correspond well to the results of the ITS model adjusted for seasonality (model 3). Figures 4, 5 show the de-seasonalized trend as well as trends produced from the ITS model 3 for COPD (all codes) and IHD (strict definition), respectively.

## Discussion

This time-trend analysis aimed to replicate our previous RWD analysis assessing the impact of HTP introduction into the Japanese market using data from MDV. This second analysis conducted using JMDC data, confirmed that HTPs' introduction in the Japanese market was associated with a reduction in hospitalizations due to COPD and IHD. That is, we detected a significant reduction in the number of hospitalizations for COPD (all codes) when using all available data from the JMDC database, and for IHD when using the DPC claims data. Additionally, when analyzing all claims data, a non-significant reduction was identified for hospitalizations due to COPD exacerbation plus LRTIs and IHD after HTPs' introduction in Japan.

Analyses of both databases showed similar results even if the pre-HTP trends were not always comparable. This could be because of the differences between the demographic characteristics of the two database populations. It should be noted that there are differences between the MDV and JMDC datasets. MDV covers ~24% of the Japanese population compared with the 8% covered by JMDC. Additionally, MDV includes a much higher percentage of people aged over 65 years (35 vs. 2% for both datasets, respectively). Moreover, the JMDC dataset does not include people aged over 75 years. These differences might explain some of the discrepancies observed between the two analyses.

This study comes with some limitations. For instance, we considered age, sex, quarterly seasonality, and flu vaccination as potential confounders; we did not include additional factors influencing the hospitalization pattern, such as legislation and policy changes. This is because, according to a recent analysis on the impact of HTP commercialization on cigarette sales (1), Japan was still implementing (in mid-2018) its first national smoke-free legislation in phases, which would last until April 2020 (8).

Additionally, when interpreting these results, it should be considered that time-trend analyses such as the one conducted here do not assess causal relationships between exposure and outcomes at the individual level; rather, they evaluate the potential impact of a population-based intervention on a population's health (9). Such analyses, however, are important in the context of epidemiological and public health research as well as for hypothesis generation (10). For instance, many diseases show remarkable fluctuations in incidence over time; in particular, chronic disorders such as lung cancer, COPD, and coronary heart disease evolve over many years. If time or secular trends in disease incidence correlate with changes in a community's environment or way of life, then the trends may provide important clues about disease etiology (10).

This study's findings provide insights into the potential impact of HTP commercialization on the hospitalizations associated with COPD and IHD. Regarding the impact of HTP commercialization on hospitalizations for other smoking-related endpoints, a longer post-HTP introduction follow-up might be needed. As a next step, these findings warrant further research to evaluate the impact of HTPs on the health of populations in other countries where these products have been introduced. Despite the study's limitations, findings from these studies provide important insights on the potential health impacts of HTPs before long-term epidemiological data becomes available.

## Data Availability Statement

The data analyzed in this study is subject to the following licenses/restrictions: The data that support the findings of this study are available from JMDC. However, restrictions apply to the availability of these data, which were used under license for the current study and are, therefore, not publicly available. Requests to access these datasets should be directed to https://www.jmdc.co.jp/en/inquiry/.

## Author Contributions

AP and MH contributed to study conception and design as well as data acquisition and interpretation. MA and AR-K contributed to the analysis and manuscript writing. AH contributed to study conception and data acquisition. All authors reviewed the manuscript.

## Funding

Philip Morris International is the sole source of funding and sponsor of this research.

## Conflict of Interest

AP, MA, AR-K, MH, and AH are employees of Philip Morris International.

## Publisher's Note

All claims expressed in this article are solely those of the authors and do not necessarily represent those of their affiliated organizations, or those of the publisher, the editors and the reviewers. Any product that may be evaluated in this article, or claim that may be made by its manufacturer, is not guaranteed or endorsed by the publisher.

## Abbreviations

COPD: chronic obstructive pulmonary disease
DPC: diagnosis procedure combination
HTP: heated tobacco product
IHD: ischemic heart disease
ITS: interrupted time-series
JMDC: Japanese Medical Data Center
LRTI: lower respiratory tract infection
MDV: medical data vision
PMI: Philip Morris international
RWD: real-world data
RWE: real-world evidence.
