# Supplementary material for: Ischemic Heart Disease and Chronic Obstructive Pulmonary Disease Hospitalizations in Japan Before and After the Introduction of a Heated Tobacco Product
Source: Front Public Health. 2022 Jun 28;10:909459. doi: 10.3389/fpubh.2022.909459 (PMC9275563; doi:10.3389/fpubh.2022.909459)
Supplement: Supplementary file 1 [file Table_1.DOCX]

Supplementary Table 1. Results of interrupted time-series Poisson regression on number of hospitalizations due to chronic obstructive pulmonary disease (all codes).

|  |  | **Model 1: No confounder** | | **Model 2: Sex + age** | | **Model 3: Sex + age + seasonality** | | **Model 4: Sex + age + seasonality + flu vaccination** | |
| --- | --- | --- | --- | --- | --- | --- | --- | --- | --- |
|  | **Definition** | **Broad** | **Strict** | **Broad** | **Strict** | **Broad** | **Strict** | **Broad** | **Strict** |
| **Intercept** | Effect | -4.02017 | -7.93219 | -9.3157 | -25.5532 | -8.37743 | -22.45668 | -8.07894 | -21.35597 |
|  | 95% CIs | [-4.06052, -3.99215] | [-8.62255, -7.24043] | [-11.51293, -7.26443] | [-43.37666, -7.73101] | [-10.41431, -6.35387] | [-40.9906, -3.92278] | [-10.81978, -5.52396] | [-49.25597, 6.54403] |
|  | p-value | p<0.0001 | p<0.0001 | p<0.0001 | p=0.0050 | p<0.0001 | p=0.0176 | p<0.0001 | p=0.1335 |
| **Step change**  **(pre-post gap)** | Effect | 0.2951 | 0.754 | 0.1962 | 0.3753 | 0.2169 | 0.22247 | 0.2109 | 0.21499 |
|  | 95% CIs | [0.20689, 0.38336] | [-0.51, 2.01875] | [0.10444, 0.28811] | [-0.99855, 1.74915] | [0.1307, 0.30329] | [-1.16533, 1.61027] | [0.1186, 0.30335] | [-1.17978, 1.60976] |
|  | p-value | p<0.0001 | p=0.2422 | p<0.0001 | p=0.5924 | p<0.0001 | p=0.7534 | p<0.0001 | p=0.7626 |
| **Pre-HTP slope** | Effect | 0.0035 | 0.00249 | 0.0002 | -0.00914 | 0.001 | -0.00513 | 0.0011 | -0.00493 |
|  | 95% CIs | [0.00254, 0.00466] | [-0.00793, 0.0129] | [-0.00123, 0.00178] | [-0.02362, 0.00534] | [-0.00039, 0.00258] | [-0.02019, 0.00993] | [-0.00037, 0.00265] | [-0.02046, 0.01061] |
|  | p-value | p<0.0001 | p=0.6397 | p=0.7236 | p=0.2162 | p=0.1543 | p=0.5042 | p=0.1454 | p=0.5342 |
| **Trend change**  **(pre- vs. post-HTP)** | Effect | -0.00707 | -0.0064 | -0.004 | -0.0005 | -0.005 | -0.00018 | -0.005 | -0.00064 |
|  | 95% CIs | [-0.00868, -0.00549] | [-0.02097, 0.00817] | [-0.00645, -0.00267] | [-0.01647, 0.01546] | [-0.00698, -0.00338] | [-0.01633, 0.01596] | [-0.00725, -0.00337] | [-0.01891, 0.01764] |
|  | p-value | p<0.0001 | p=0.3891 | p<0.0001 | p=0.9510 | p<0.0001 | p=0.9821 | p<0.0001 | p=0.9455 |
| **Women %** | Effect |  |  | 2.2779 | 0.83662 | 2.0214 | 2.96824 | 2.0139 | 3.05306 |
|  | 95% CIs |  |  | [0.53833, 4.01818] | [-13.12715, 14.80039] | [0.31678, 3.72613] | [-10.92514, 16.88871] | [0.29936, 3.72872] | [-10.92514, 17.05657] |
|  | p-value |  |  | p=0.0122 | p=0.9065 | p=0.0228 | p=0.6760 | p=0.0242 | p=0.6692 |
| **Average age** | Effect |  |  | 0.0895 | 0.37005 | 0.0711 | 0.27623 | 0.0708 | 0.2729 |
|  | 95% CIs |  |  | [0.05917, 0.11996] | [0.07265, 0.66745] | [0.04092, 0.10142] | [-0.04198, 0.59445] | [0.04034, 0.1013] | [-0.05147, 0.59727] |
|  | p-value |  |  | p<0.0001 | p=0.0147 | p<0.0001 | p=0.0889 | p<0.0001 | p=0.0992 |
| **Spring** | Effect |  |  |  |  | 0.0404 | 0.12641 | 0.0404 | 0.12684 |
|  | 95% CIs |  |  |  |  | [0.01901, 0.06198] | [-0.11983, 0.37264] | [0.01882, 0.06205] | [-0.11957, 0.37325] |
|  | p-value |  |  |  |  | p=0.0004 | p=0.3143 | p=0.0005 | p=0.3130 |
| **Autumn** | Effect |  |  |  |  | 0.0287 | -0.14999 | 0.0289 | -0.14882 |
|  | 95% CIs |  |  |  |  | [0.00731, 0.05019] | [-0.40703, 0.10706] | [0.00733, 0.0505] | [-0.40683, 0.10918] |
|  | p-value |  |  |  |  | p<0.0001 | p= 0.2528 | p=0.0105 | p=0.2582 |
| **Winter** | Effect |  |  |  |  | 0.0329 | 0.24081 | 0.0329 | 0.24201 |
|  | 95% CIs |  |  |  |  | [0.01043, 0.05545] | [-0.01518, 0.49681] | [0.0103, 0.0556] | [-0.01504, 0.49907] |
|  | p-value |  |  |  |  | p=0.0054 | p=0.0652 | p=0.0056 | p=0.0650 |
| **Flu vaccination** | Effect |  |  |  |  |  |  | -0.54561 | -1.96764 |
|  | 95% CIs |  |  |  |  |  |  | [-3.41398, 2.3227] | [-39.26776, 35.33248] |
|  | p-value |  |  |  |  |  |  | p=0.7103 | p=0.9177 |

Note: HTP: heated tobacco product, CI: confidence interval.
