# Supplementary material for: Ischemic Heart Disease and Chronic Obstructive Pulmonary Disease Hospitalizations in Japan Before and After the Introduction of a Heated Tobacco Product
Source: Front Public Health. 2022 Jun 28;10:909459. doi: 10.3389/fpubh.2022.909459 (PMC9275563; doi:10.3389/fpubh.2022.909459)
Supplement: Supplementary file 2 [file Table_2.DOCX]

Supplementary Table 2. Results of interrupted time-series Poisson regression on number of hospitalizations due to chronic obstructive pulmonary disease exacerbations.

|  |  | **Model 1: No confounder** | | **Model 2: Sex + age** | | **Model 3: Sex + age + seasonality** | | **Model 4: Sex + age + seasonality + flu vaccination** | |
| --- | --- | --- | --- | --- | --- | --- | --- | --- | --- |
|  | **Definition** | **Broad** | **Strict** | **Broad** | **Strict** | **Broad** | **Strict** | **Broad** | **Strict** |
| **Intercept** | Effect | -9.21034 | -10.10194 | -29.9009 | -35.93159 | -25.70568 | -30.78931 | -13.81551 | -21.61886 |
|  | 95% CIs | [-9.72117, -8.74034] | [-11.3306, -8.77209] | [-56.14243, -3.65932] | [-65.5701, -6.29311] | [-51.73307, 0.32172] | [-61.7774, 0.19877] | [-49.26244, 21.78887] | [-70.52328, 27.28556] |
|  | p-value | p<0.0001 | p<0.0001 | p=0.0284 | p=0.0175 | p=0.0567 | p=0.0515 | p=0.4509 | p= 0.3863 |
| **Step change**  **(pre-post gap)** | Effect | -0.244 | -1.75443 | -0.433 | -2.30797 | -0.25268 | -2.48825 | -0.5495 | -2.62812 |
|  | 95% CIs | [-1.13289, 0.64337] | [-3.96949, 0.4606] | [-1.49781, 0.63138] | [-4.67046, 0.05452] | [-1.28877, 0.7834] | [-4.88013, -0.09646] | [-1.73642, 0.63739] | [-5.0787, -0.17765] |
|  | p-value | p=0.5905 | p=0.1206 | p=0.4276 | p=0.0555 | p=0.6340 | p=0.0414 | p=0.3671 | p= 0.0355 |
| **Pre-HTP slope** | Effect | 0.0125 | 0.01839 | 0.0029 | 0.00167 | 0.0115 | 0.00762 | 0.0124 | 0.00865 |
|  | 95% CIs | [-0.0017, 0.02689] | [-0.00091, 0.03769] | [-0.01774, 0.02358] | [-0.02408, 0.02741] | [-0.00913, 0.03221] | [-0.01939, 0.03464] | [-0.00795, 0.03283] | [-0.01838, 0.03567] |
|  | p-value | p=0.0891 | p=0.0616 | p=0.7828 | p=0.8990 | p=0.2774 | p=0.5803 | p=0.2358 | p=0.5306 |
| **Trend change**  **(pre- vs. post-HTP)** | Effect | 0.0078 | 0.01417 | 0.013 | 0.02266 | 0.00616 | 0.02278 | 0.0022 | 0.01999 |
|  | 95% CIs | [-0.0099, 0.02571] | [-0.0112, 0.03954] | [-0.01047, 0.03649] | [-0.00464, 0.04995] | [-0.01685, 0.02917] | [-0.00495, 0.0505] | [-0.02188, 0.02634] | [-0.00991, 0.04989] |
|  | p-value | p=0.3901 | p=0.2736 | p=0.2810 | p=0.1038 | p=0.6015 | p=0.1074 | p=0.8567 | p= 0.1900 |
| **Women %** | Effect |  |  | 12.337 | 6.04706 | 15.159 | 6.03774 | 14.462 | 6.65584 |
|  | 95% CIs |  |  | [-56.14243, 34.53878] | [-17.20115, 29.29526] | [-6.42102, 36.736] | [-17.26925, 29.34473] | [-7.05858, 35.92507] | [-16.68533, 29.99701] |
|  | p-value |  |  | p=0.2826 | p=0.6102 | p=0.1727 | p=0.6116 | p=0.3998 | p= 0.5762 |
| **Average age** | Effect |  |  | 0.3101 | 0.49534 | 0.18 | 0.37706 | 0.169 | 0.35136 |
|  | 95% CIs |  |  | [-0.07518, 0.69556] | [-0.01259, 1.00327] | [-0.21072, 0.57087] | [-0.16803, 0.92215] | [-0.22212, 0.56017] | [-0.20311, 0.90583] |
|  | p-value |  |  | p=0.1187 | p=0.0560 | p=0.3694 | p=0.1752 | p=0.3998 | p=0.2142 |
| **Spring** | Effect |  |  |  |  | 0.2255 | 0.35478 | 0.21988 | 0.35495 |
|  | 95% CIs |  |  |  |  | [-0.03341, 0.48459] | [-0.03506, 0.74462] | [-0.04015, 0.47992] | [-0.03533, 0.74522] |
|  | p-value |  |  |  |  | p=0.0919 | p=0.0745 | p=0.1017 | p=0.0747 |
| **Autumn** | Effect |  |  |  |  | -0.029 | -0.09362 | -0.02 | -0.08383 |
|  | 95% CIs |  |  |  |  | [-0.29244, 0.23311] | [-0.49928, 0.31205] | [-0.28489, 0.24304] | [-0.49156, 0.3239] |
|  | p-value |  |  |  |  | p=0.825474 | p=0.6511 | p=0.8770 | p=0.6870 |
| **Winter** | Effect |  |  |  |  | 0.325 | 0.23786 | 0.324 | 0.24513 |
|  | 95% CIs |  |  |  |  | [0.0624, 0.58776] | [-0.17538, 0.65109] | [0.0602, 0.58785] | [-0.16974, 0.66] |
|  | p-value |  |  |  |  | p=0.01769 | p=0.2593 | p=0.0186 | p=0.2468 |
| **Flu vaccination** | Effect |  |  |  |  |  |  | -22.05766 | -16.42089 |
|  | 95% CIs |  |  |  |  |  |  | [-66.75237, 22.63705] | [-84.44437, 51.60259] |
|  | p-value |  |  |  |  |  |  | p=0.3366 | p=0.6361 |

Note: HTP: heated tobacco product, CI: confidence interval.
