# Supplementary material for: Ischemic Heart Disease and Chronic Obstructive Pulmonary Disease Hospitalizations in Japan Before and After the Introduction of a Heated Tobacco Product
Source: Front Public Health. 2022 Jun 28;10:909459. doi: 10.3389/fpubh.2022.909459 (PMC9275563; doi:10.3389/fpubh.2022.909459)
Supplement: Supplementary file 3 [file Table_3.DOCX]

Supplementary Table 3. Results of interrupted time-series Poisson regression on number of hospitalizations due to chronic obstructive pulmonary disease exacerbations + lower respiratory tract infection.

|  |  | **Model 1: No confounder** | | **Model 2: Sex + age** | | **Model 3: Sex + age + seasonality** | | **Model 4: Sex + age + seasonality + flu vaccination** | |
| --- | --- | --- | --- | --- | --- | --- | --- | --- | --- |
|  | **Definition** | **Broad** | **Strict** | **Broad** | **Strict** | **Broad** | **Strict** | **Broad** | **Strict** |
| **Intercept** | Effect | -5.50904 | -10.38152 | -16.02457 | -32.89185 | -14.63873 | -28.1985 | -15.99947 | -8.55802 |
|  | 95% CIs | [-5.61577, -5.41485] | [-11.69718, -9.03457] | [-22.77089, -9.3157] | [-61.45041, -4.33331] | [-20.77811, -8.51719] | [-58.15196, 1.75496] | [-23.78235, -8.21709] | [-56.17717, 39.06616] |
|  | p-value | p<0.0001 | p<0.0001 | p<0.0001 | p=0.02399 | p<0.0001 | p=0.06502 | p=0.0001 | p=0.7248 |
| **Step change**  **(pre-post gap)** | Effect | 0.1497 | -1.10493 | -0.075 | -1.63989 | 0.0238 | -1.8153 | 0.0514 | -2.1331 |
|  | 95% CIs | [-0.11535, 0.41479] | [-3.26317, 1.05331] | [-0.37753, 0.22644] | [-3.94755, 0.66778] | [-0.24079, 0.28846] | [-4.15142, 0.52083] | [-0.23165, 0.33453] | [-4.52055, 0.25436] |
|  | p-value | p=0.2716 | p=0.31566 | p=0.6253 | p=0.16368 | p=0.8603 | p=0.12776 | p=0.7228 | p=0.0799 |
| **Pre-HTP slope** | Effect | 0.0035 | 0.02315 | -0.003 | 0.00878 | 0.0003 | 0.01479 | 0.00009 | 0.01657 |
|  | 95% CIs | [0.00044, 0.00671] | [0.00404, 0.04227] | [-0.00847, 0.00137] | [-0.01664, 0.03419] | [-0.0042, 0.00482] | [-0.01195, 0.04152] | [-0.00451, 0.0047] | [-0.00967, 0.04282] |
|  | p-value | p=0.0282 | p=0.01761 | p=0.1609 | p=0.49861 | p=0.8941 | p=0.27837 | p=0.9688 | p=0.2158 |
| **Trend change**  **(pre- vs. post-HTP)** | Effect | -0.005 | 0.00637 | 0.0003 | 0.01417 | -0.003 | 0.01416 | -0.002 | 0.0085 |
|  | 95% CIs | [-0.01012, -0.00057] | [-0.01845, 0.0312] | [-0.0058, 0.00655] | [-0.01262, 0.04095] | [-0.00853, 0.00246] | [-0.01307, 0.04139] | [-0.00835, 0.00349] | [-0.0205, 0.0375] |
|  | p-value | 0.0312 | p=0.61475 | p=0.9071 | p=0.29988 | p=0.2833 | p=0.30798 | p=0.4237 | p=0.5656 |
| **Women** | Effect |  |  | 3.9804 | 7.04769 | 5.81329 | 7.30656 | 5.84432 | 8.54677 |
| **%** | 95% CIs |  |  | [-1.74177, 9.70267] | [-15.40537, 29.50075] | [5.79773, 5.82865] | [-15.23805, 29.85117] | [5.82911, 5.8593] | [-13.90106, 30.9946] |
|  | p-value |  |  | p=0.1767 | p=0.53842 | p=0.0308 | p=0.52529 | p=0.0311 | p=0.4555 |
| **Average age** | Effect |  |  | 0.1847 | 0.41469 | 0.1307 | 0.30253 | 0.1326 | 0.25013 |
|  | 95% CIs |  |  | [0.08444, 0.28516] | [-0.07585, 0.90522] | [0.03836, 0.2232] | [-0.22521, 0.83027] | [0.03948, 0.2259] | [-0.28521, 0.78547] |
|  | p-value |  |  | p=0.0005 | p=0.09754 | p=0.0070 | p=0.2612 | p=0.0067 | p=0.3598 |
| **Spring** | Effect |  |  |  |  | 0.0958 | 0.33356 | 0.096 | 0.33367 |
|  | 95% CIs |  |  |  |  | [0.03006, 0.16156] | [-0.04191, 0.70902] | [0.02991, 0.1621] | [-0.04274, 0.71008] |
|  | p-value |  |  |  |  | p=0.0055 | p=0.081651 | p=0.0057 | p=0.0823 |
| **Autumn** | Effect |  |  |  |  | -0.018 | -0.13575 | -0.019 | -0.1153 |
|  | 95% CIs |  |  |  |  | [-0.08513, 0.04847] | [-0.5282, 0.25671] | [-0.08627, 0.04814] | [-0.5098, 0.27919] |
|  | p-value |  |  |  |  | p=0.5922 | p=0.49781 | p=0.5798 | p=0.5667 |
| **Winter** | Effect |  |  |  |  | 0.141 | 0.21895 | 0.1409 | 0.23331 |
|  | 95% CIs |  |  |  |  | [0.07321, 0.20887] | [-0.17955, 0.61744] | [0.07273, 0.20911] | [-0.16733, 0.63394] |
|  | p-value |  |  |  |  | p=0.0001 | p=0.281536 | p=0.0001 | p=0.2537 |
| **Flu vaccination** | Effect |  |  |  |  |  |  | 2.50402 | -35.30385 |
|  | 95% CIs |  |  |  |  |  |  | [1.25831, 3.04184] | [-102.40692, 31.79923] |
|  | p-value |  |  |  |  |  |  | p=0.5749 | p=0.3025 |

Note: HTP: heated tobacco product, CI: confidence interval.
