# Supplementary material for: Ischemic Heart Disease and Chronic Obstructive Pulmonary Disease Hospitalizations in Japan Before and After the Introduction of a Heated Tobacco Product
Source: Front Public Health. 2022 Jun 28;10:909459. doi: 10.3389/fpubh.2022.909459 (PMC9275563; doi:10.3389/fpubh.2022.909459)
Supplement: Supplementary file 4 [file Table_4.DOCX]

Supplementary Table 4. Results of interrupted time-series Poisson regression on number of hospitalizations due to ischemic heart disease.

|  |  | **Model 1: No confounder** | | **Model 2: Sex + age** | | **Model 3: Sex + age + seasonality** | | **Model 4: Sex + age + seasonality + flu vaccination** | |
| --- | --- | --- | --- | --- | --- | --- | --- | --- | --- |
|  | **Definition** | **Broad** | **Strict** | **Broad** | **Strict** | **Broad** | **Strict** | **Broad** | **Strict** |
| **Intercept** | Effect | -3.13109 | -3.77661 | -5.61577 | -7.26158 | -5.29035 | -6.86661 | -5.58867 | -8.06612 |
|  | 95% CIs | [-3.16297, -3.10087] | [-3.86062, -3.69261] | [-7.6009, -3.63515] | [-9.53884, -4.98001] | [-7.40205, -3.19883] | [-9.26163, -4.46619] | [-8.25483, -2.93332] | [-11.51293, -4.61028] |
|  | p-value | p<0.0001 | p<0.0001 | p<0.0001 | p<0.0001 | p<0.0001 | p<0.0001 | p<0.0001 | p<0.0001 |
| **Step change**  **(pre-post gap)** | Effect | 0.2195 | 0.84198 | 0.0926 | 0.74902 | 0.0933 | 0.74683 | 0.099 | 0.7565 |
|  | 95% CIs | [0.13645, 0.30255] | [0.67362, 1.01033] | [0.00315, 0.18225] | [0.56418, 0.93386] | [0.00268, 0.18394] | [0.56024, 0.93342] | [0.00251, 0.19554] | [0.56884, 0.9442] |
|  | p-value | p<0.0001 | p<0.0001 | p=0.04 | p<0.0001 | p=0.0472 | p<0.0001 | p=0.0480 | p<0.0001 |
| **Pre-HTP slope** | Effect | 0.0005 | 13.81912 | -0.002 | 0.00139 | -0.0024 | 0.00175 | -0.0024 | 0.00153 |
|  | 95% CIs | [-0.0004, 0.00156] | [0.00234, 0.00487] | [-0.00387, -0.00096] | [-0.00046, 0.00323] | [-0.00399, -0.00089] | [-0.00017, 0.00367] | [-0.00407, -0.0009] | [-0.00045, 0.0035] |
|  | p-value | p=0.2537 | p<0.001 | p=0.0017 | p=0.1400 | p=0.0029 | p=0.0744 | p=0.0029 | p=0.1297 |
| **Trend change**  **(pre- vs. post-HTP)** | Effect | -0.0045 | -0.01026 | -0.001 | -0.00897 | -0.00137 | -0.00904 | -0.001 | -0.00854 |
|  | 95% CIs | [-0.00606, -0.00305] | [-0.01215, -0.00837] | [-0.00325, 0.00042] | [-0.01107, -0.00686] | [-0.00325, 0.00051] | [-0.01116, -0.00692] | [-0.00327, 0.00081] | [-0.0109, -0.00619] |
|  | p-value | p<0.0001 | p<0.001 | p=0.1337 | p<0.0001 | p=0.1595 | p<0.0001 | p=0.2406 | p<0.0001 |
| **Women %** | Effect |  |  | -0.42599 | 1.00397 | -0.74001 | 1.16871 | -0.73599 | 1.0317 |
|  | 95% CIs |  |  | [-2.11478, 1.26094] | [-0.8258, 2.83375] | [-2.52585, 1.04222] | [-0.67557, 3.01299] | [-2.53112, 1.05874] | [-0.83554, 2.89894] |
|  | p-value |  |  | p=0.6215 | p=0.2822 | p=0.4177 | p=0.2142 | p=0.4240 | p=0.2788 |
| **Average age** | Effect |  |  | 0.06 | 0.065 | 0.0567 | 0.0546 | 0.0571 | 0.05812 |
|  | 95% CIs |  |  | [0.03051, 0.08958] | [0.0272, 0.10281] | [0.02516, 0.08841] | [0.01364, 0.09556] | [0.02528, 0.08904] | [0.01651, 0.09973] |
|  | p-value |  |  | p=0.0001 | p=0.00075 | p=0.0007 | p=0.0090 | p=0.0008 | p=0.0062 |
| **Spring** | Effect |  |  |  |  | -0.009 | -0.00312 | -0.0098 | -0.00401 |
|  | 95% CIs |  |  |  |  | [-0.03257, 0.0127] | [-0.03569, 0.02945] | [-0.03267, 0.01288] | [-0.03662, 0.02861] |
|  | p-value |  |  |  |  | p=0.3923 | p=0.8510 | p=0.3970 | p=0.8097 |
| **Autumn** | Effect |  |  |  |  | 0.0059 | -0.00459 | 0.0058 | -0.00585 |
|  | 95% CIs |  |  |  |  | [-0.01656, 0.02853] | [-0.03636, 0.02718] | [-0.01687, 0.02852] | [-0.03772, 0.02603] |
|  | p-value |  |  |  |  | p=0.6047 | p=0.77713 | p=0.6165 | p=0.7192 |
| **Winter** | Effect |  |  |  |  | 0.0082 | 0.03284 | 0.0081 | 0.03116 |
|  | 95% CIs |  |  |  |  | [-0.01543, 0.03187] | [-0.00172, 0.0674] | [-0.01559, 0.03197] | [-0.00357, 0.06589] |
|  | p-value |  |  |  |  | p=0.4981 | p=0.0625 | p=0.5017 | p=0.0786 |
| **Flu vaccination** | Effect |  |  |  |  |  |  | 0.53974 | 2.19833 |
|  | 95% CIs |  |  |  |  |  |  | [-2.45143, 3.53092] | [-2.3543, 6.75096] |
|  | p-value |  |  |  |  |  |  | p=0.7246 | p=0.3439 |

Note: HTP: heated tobacco product, CI: confidence interval.
